# Supplementary material for: Sulcal morphology of posteromedial cortex substantially differs between humans and chimpanzees
Source: Commun Biol. 2023 Jun 1;6:586. doi: 10.1038/s42003-023-04953-5 (PMC10235074; doi:10.1038/s42003-023-04953-5)
Supplement: Supplementary file 2 — Supplementary Information [file 42003_2023_4953_MOESM2_ESM.pdf]

# Supplementary Information

# Title: *Sulcal morphology of posteromedial cortex substantially differs between humans and chimpanzees*

Willbrand, Maboudian *et al.* (2023)

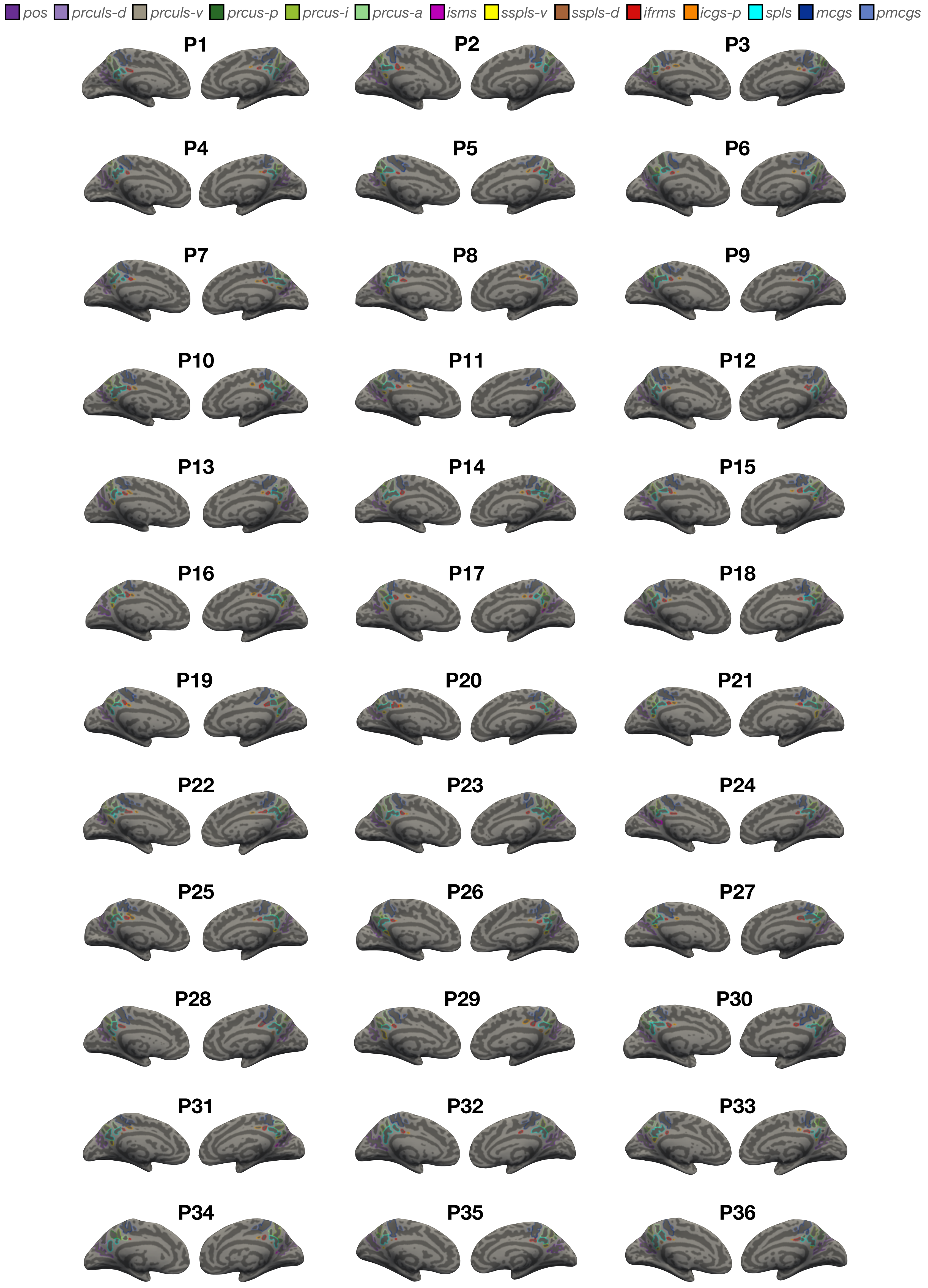
pos
prculs-d
prculs-v
prcus-p
prcus-i
prcus-a
isms
sspls-v
sspls-d
ifrms
icgs-p
spls
mcgs
pmcgs
P1
P2
P3
P4
P5
P6
P7
P8
P9
P10
P11
P12
P13
P14
P15
P16
P17
P18
P19
P20
P21
P22
P23
P24
P25
P26
P27
P28
P29
P30
P31
P32
P33
P34
P35
P36

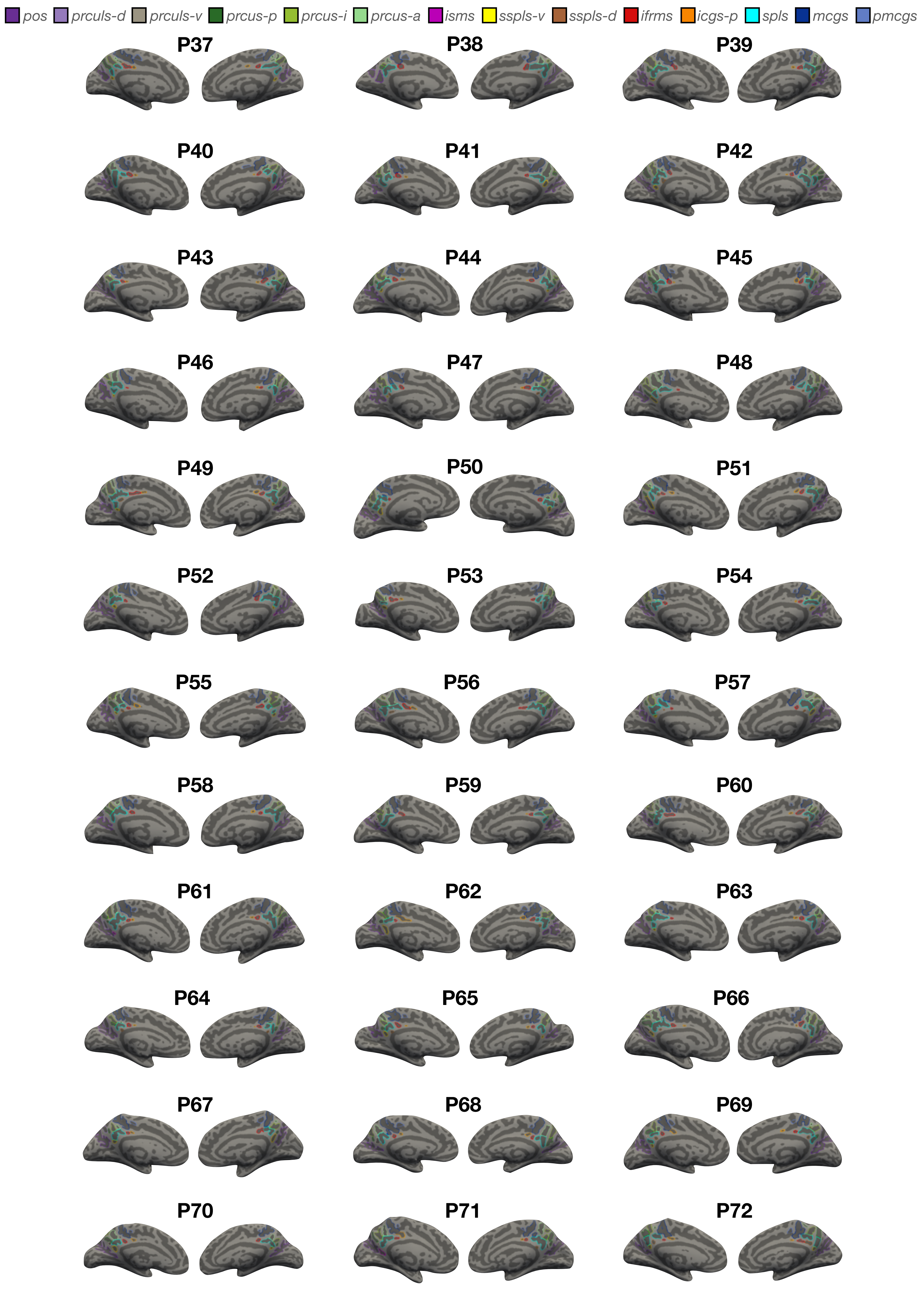


**Supplementary Figure 1. Manual PMC sulcal labels in every human participant.** Each sulcus is displayed on the left and right hemisphere inflated cortical surfaces in FreeSurfer 6.0.0, with label displayed as an outline according to the key at the top. Each hemisphere contains at least 8 sulci (from posterior to anterior): pos, prculs-d, prcus-p, prcus-i, prcus-a, spls, mcgs, and ifrms. An additional 6 sulci are variably present: isms, prculs-v, sspls-v, sspls-d, icgs-p, and pmcgs.

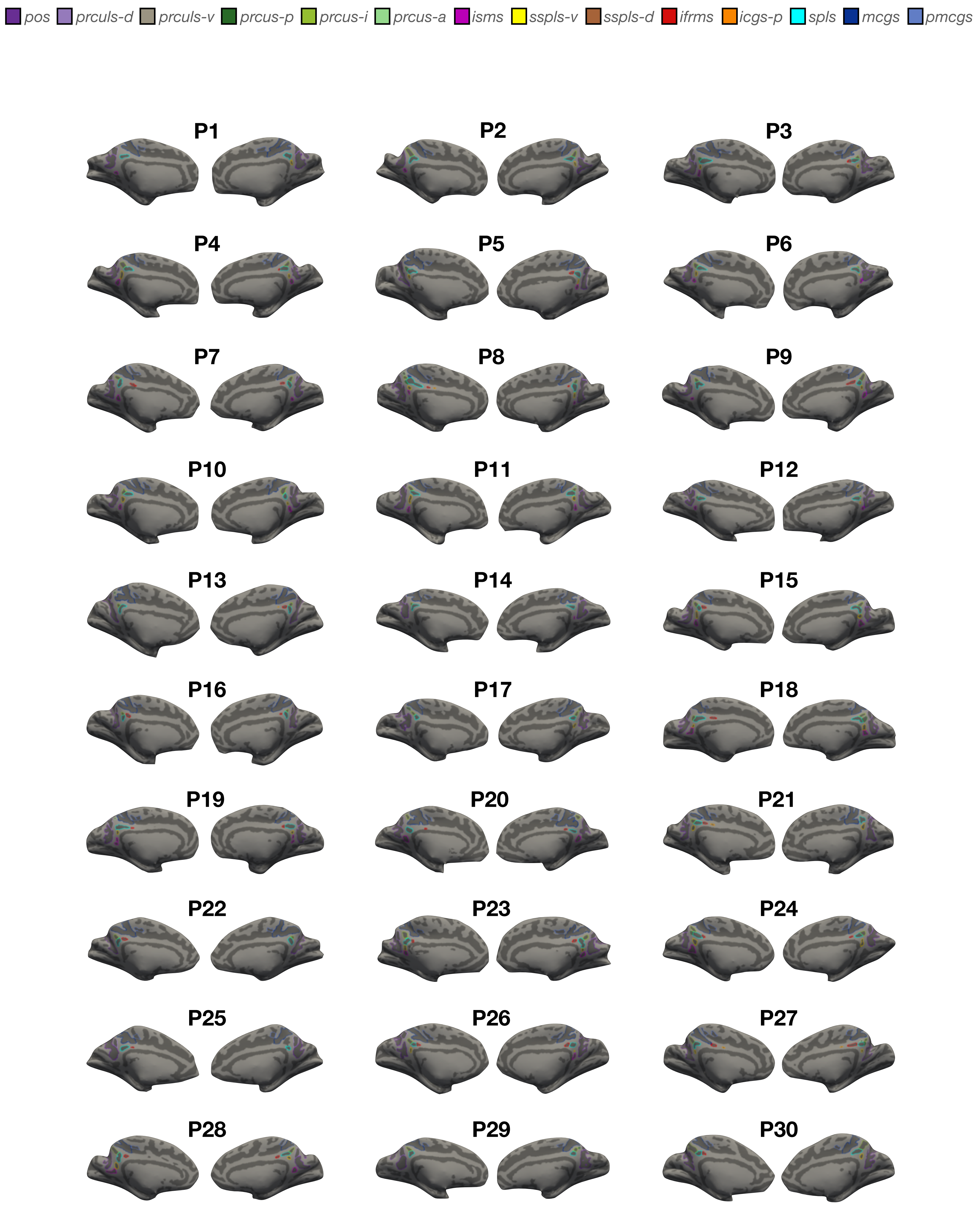
pos
prculs-d
prculs-v
prcus-p
prcus-i
prcus-a
isms
sspls-v
sspls-d
ifrms
icgs-p
spls
mcgs
pmcgs
P1
P2
P3
P4
P5
P6
P7
P8
P9
P10
P11
P12
P13
P14
P15
P16
P17
P18
P19
P20
P21
P22
P23
P24
P25
P26
P27
P28
P29
P30

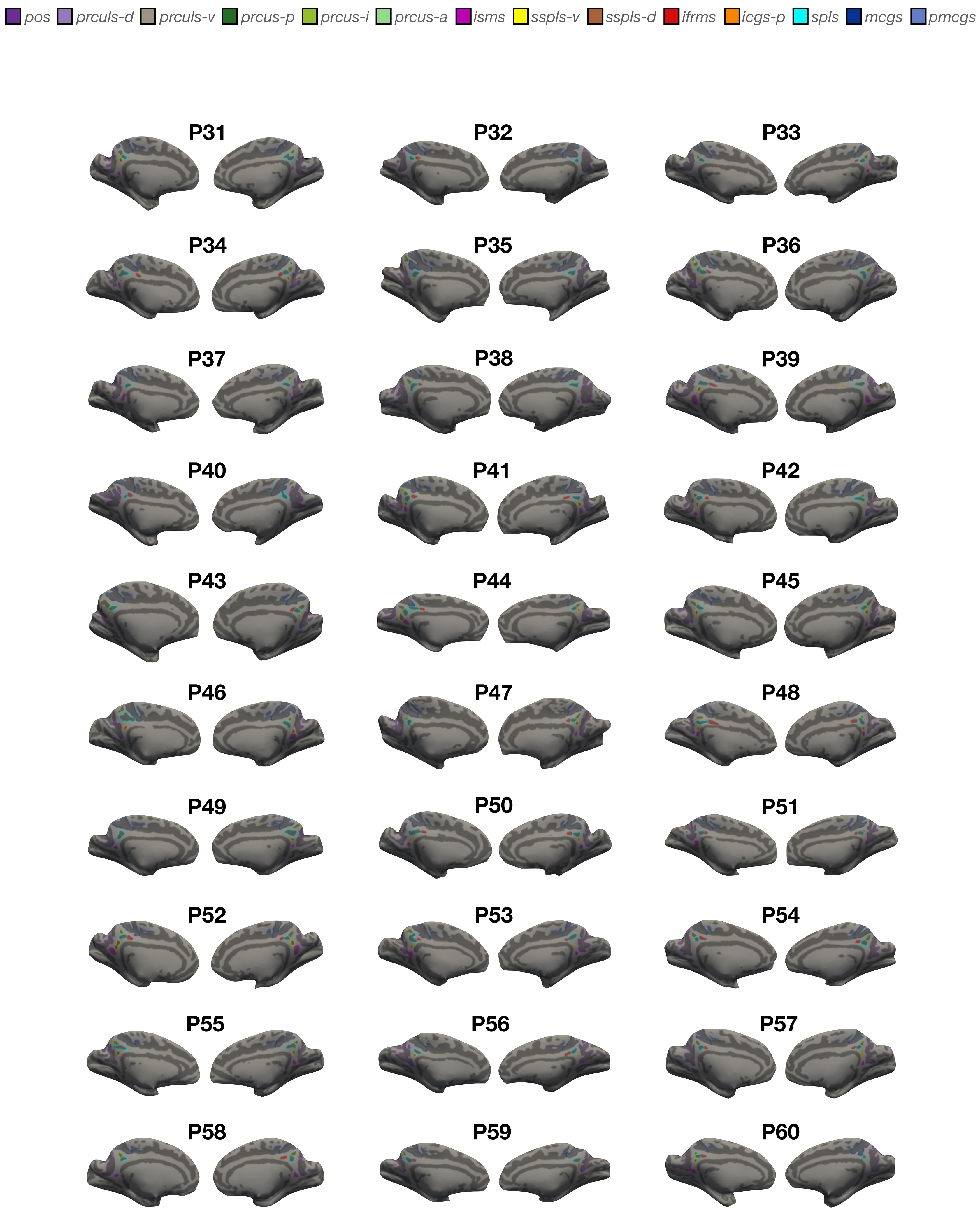


**Supplementary Figure 2. Manual PMC sulcal labels in every chimpanzee.** Each sulcus is displayed on the left and right hemisphere inflated cortical surfaces in FreeSurfer 6.0.0, with label displayed as an outline according to the key at the top. Each hemisphere contains at least 4 sulci (from posterior to anterior): mcgs, pmcgs, spls, and pos. An additional 8 sulci are variably present: prculs-d, prculs-p, prcus-i, prcus-a, isms, sspls-v, ifrms, and icgs-p. (2 sulci present in humans are not present in any chimp hemispheres: prculs-v, sspls-d).

*Newly-characterized sulci depicted, unlabeled (human)*

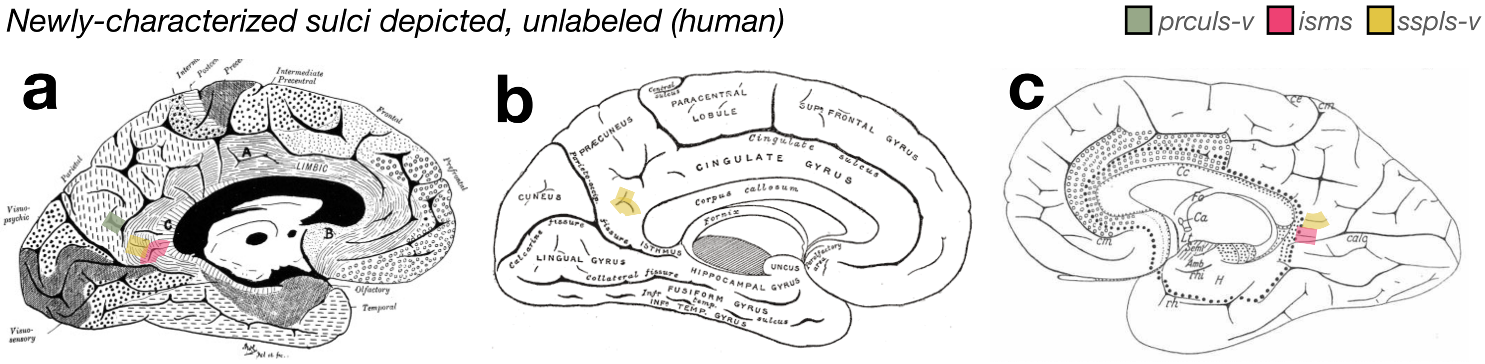


*Campbell (1905)*[1] *Gray (1918)*[2] *Vogt (1919)*[3]

**Supplementary Figure 3. Previous depictions of newly-characterized indentations by anatomists.** While we label and quantify the incidence rates of four sulci (prculs-v, isms, sspls-v, and pmcgs) across species for the first time, some classical and modern anatomists have included an unlabeled sulcus[1-6] or dimple[2,7] in the location of some of these sulci in their schematics of human brains. **a-c.** Example schematics adapted from classic sources[1-3] showing the newly-characterized sulci depicted but unlabeled in humans (images are in the public domain: https://www.law.cornell.edu/uscode/text/17/). In some modern studies, sspls-v has been labeled as part of the ventral or posterior branch of spls[8,9]. The sspls-v has also been depicted unlabeled in chimpanzee brains[10]. In all images, colored shading has been added to show the sulcal label used for each indentation in the present study.

*pos* *prculs-d* *prculs-v* *prcus-p* *prcus-i* *prcus-a* *isms* *sspls-v* *sspls-d* *ifrms* *icgs-p* *spls* *mcgs* *pmcgs*

*Nbr 3*

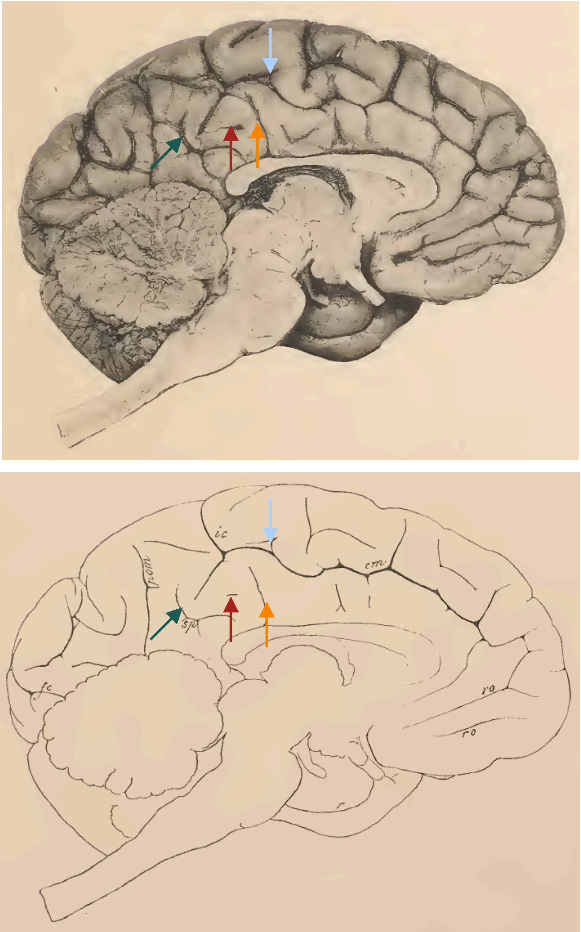


*Nbr 3*

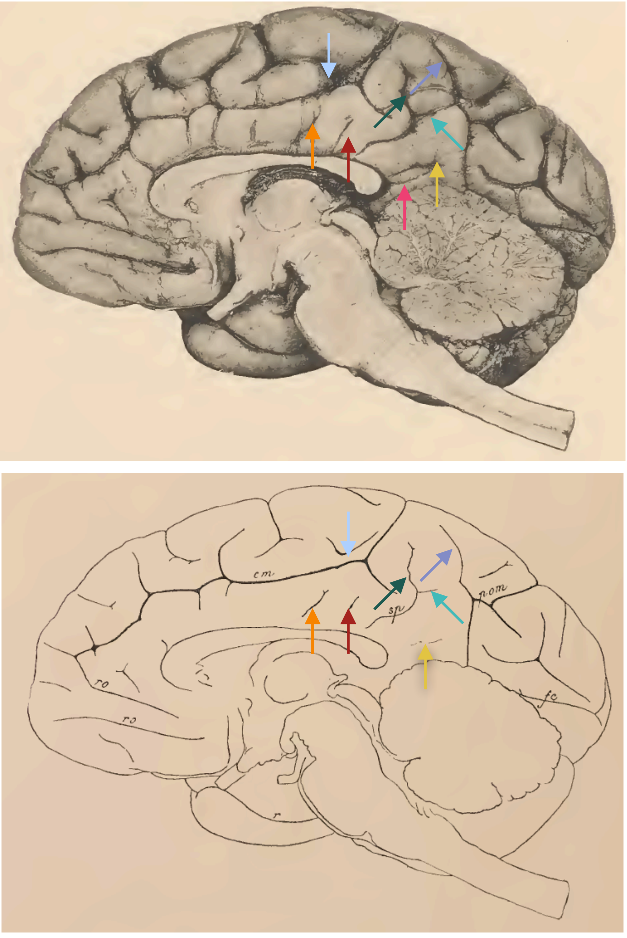


*Nbr 4*

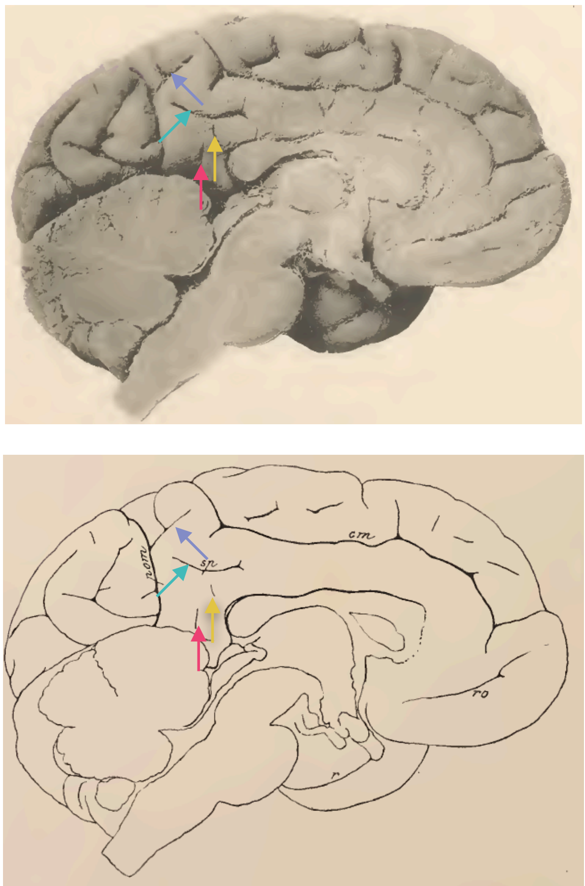


*Nbr 4*

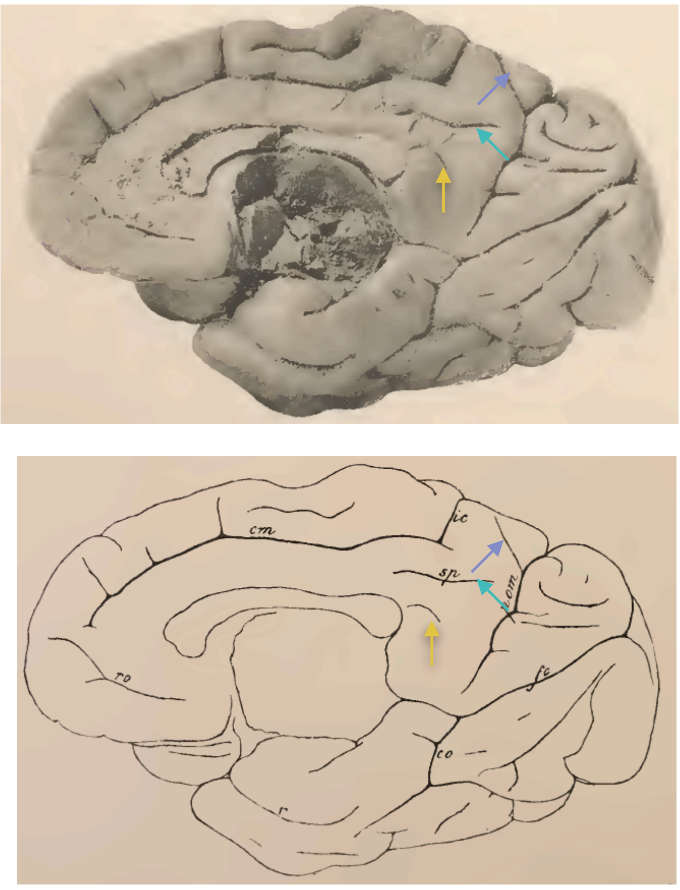

pos · prculs-d · prculs-v · prcus-p · prcus-i · prcus-a · isms · sspls-v · sspls-d · ifrms · icgs-p · spls · mcgs · pmcgs

*Nbr 5* *Nbr 5*

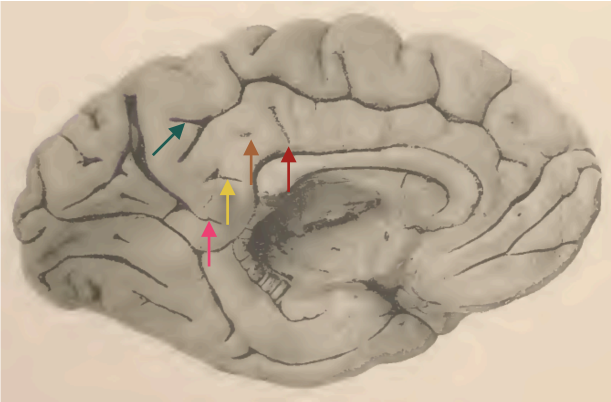
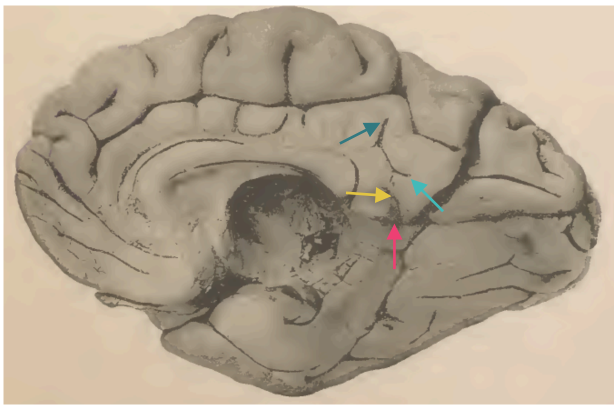
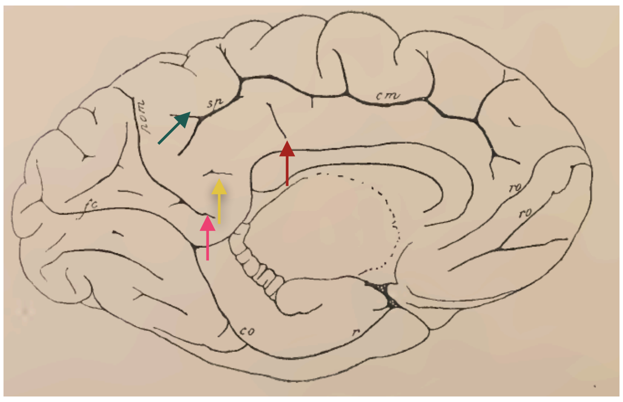

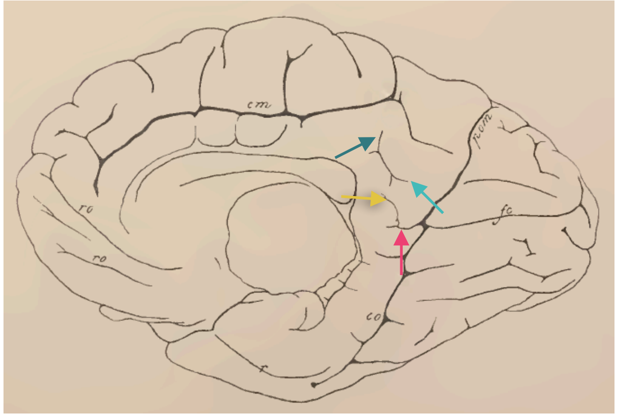


*Nbr 6* *Nbr 6*

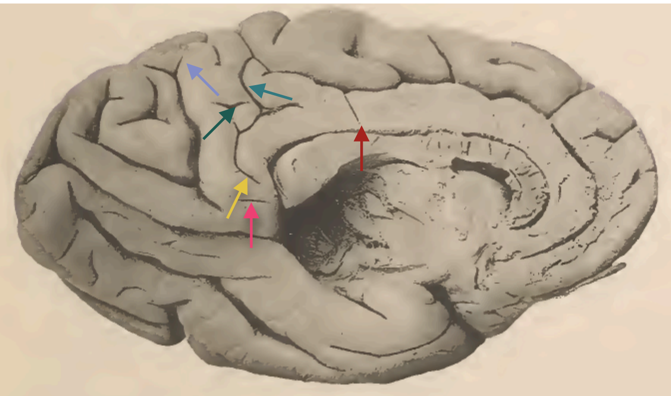
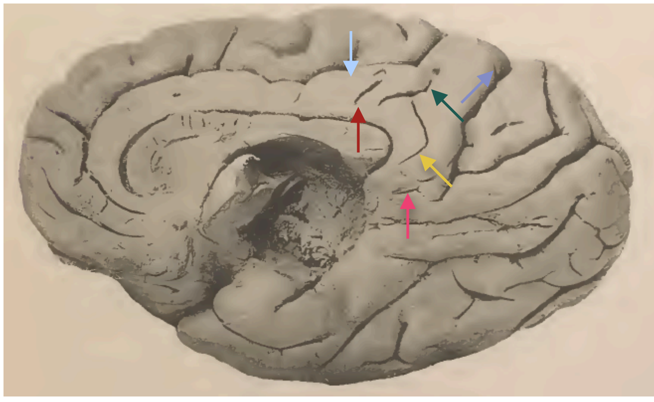
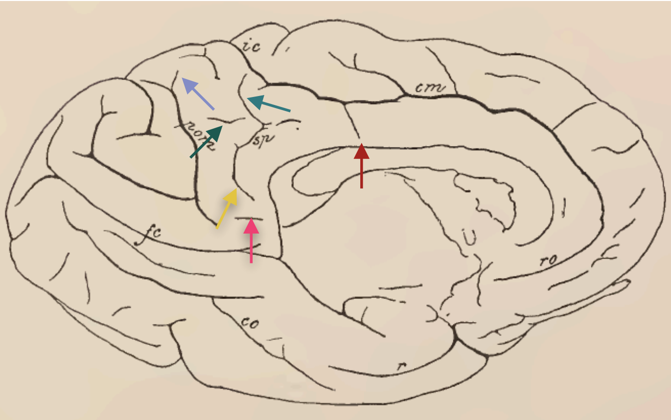

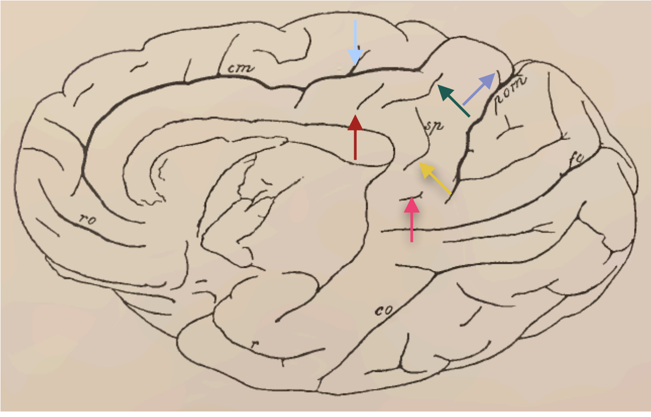

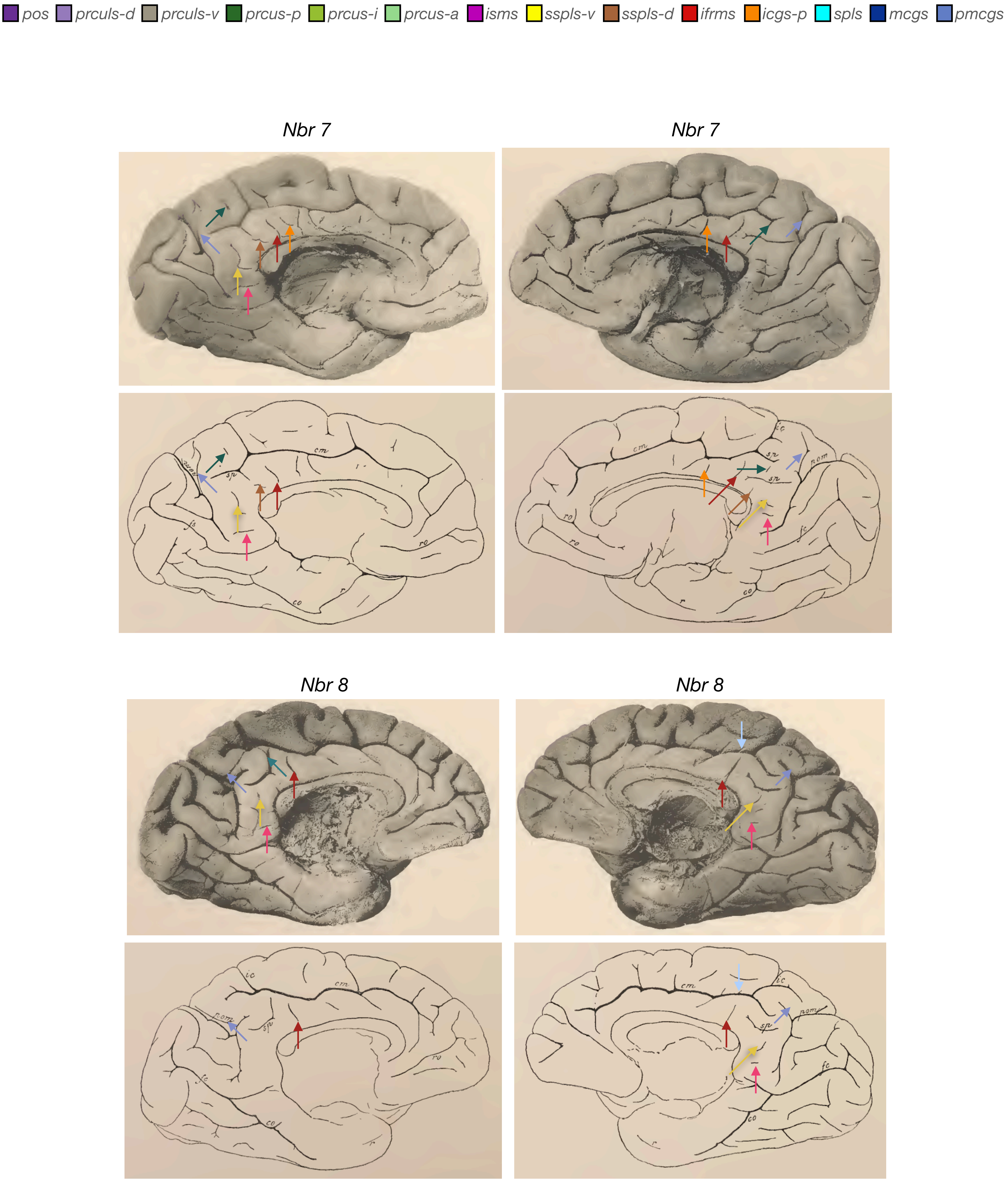
pos
prculs-d
prculs-v
prcus-p
prcus-i
prcus-a
isms
sspls-v
sspls-d
ifrms
icgs-p
spls
mcgs
pmcgs
Nbr 7
Nbr 7
Nbr 8
Nbr 8

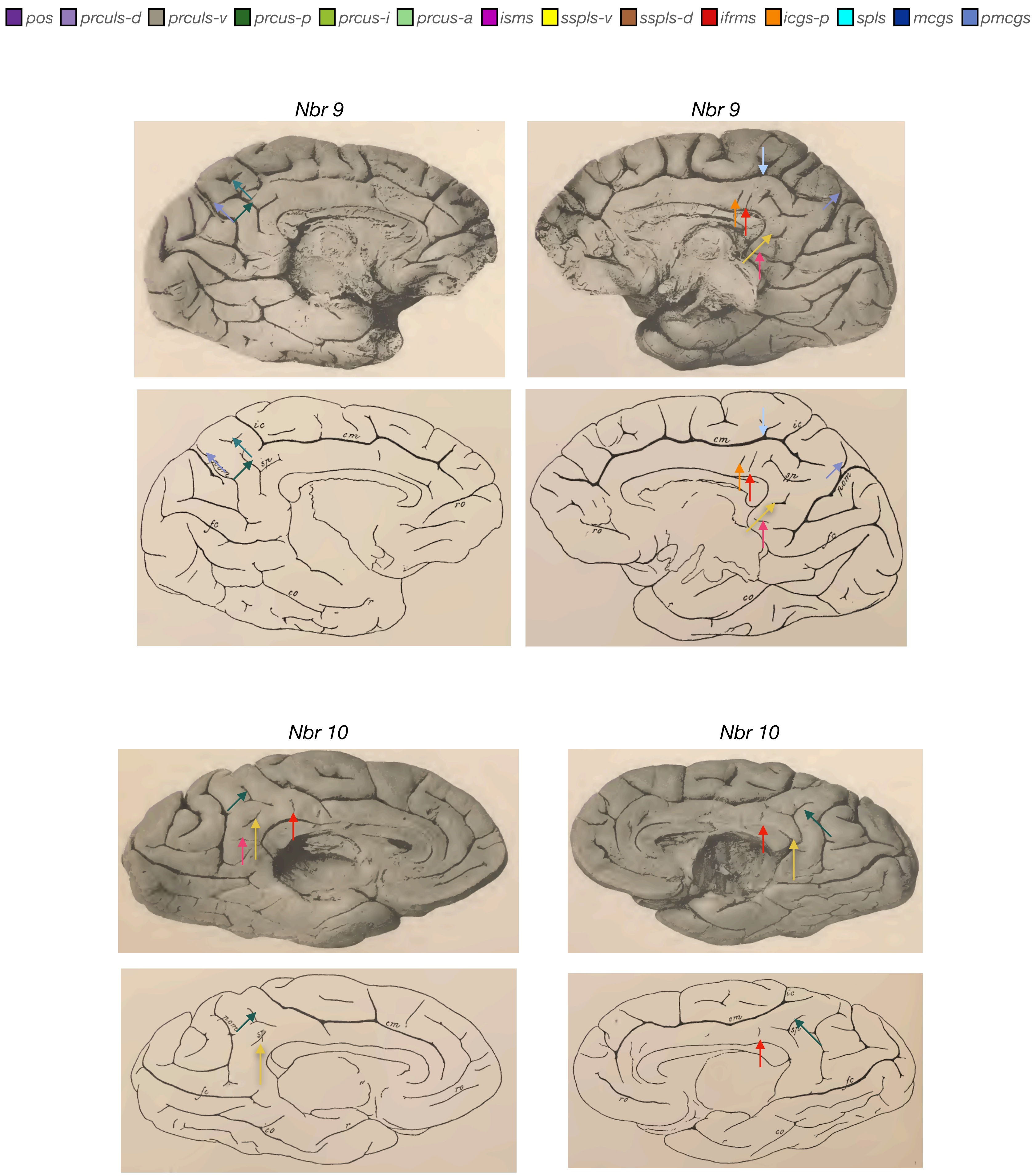
pos
prculs-d
prculs-v
prcus-p
prcus-i
prcus-a
isms
sspls-v
sspls-d
ifrms
icgs-p
spls
mcgs
pmcgs
Nbr 9
Nbr 9
Nbr 10
Nbr 10

pos prculs-d prculs-v prcus-p prcus-i prcus-a isms sspls-v sspls-d ifrms icgs-p spls mcgs pmcgs

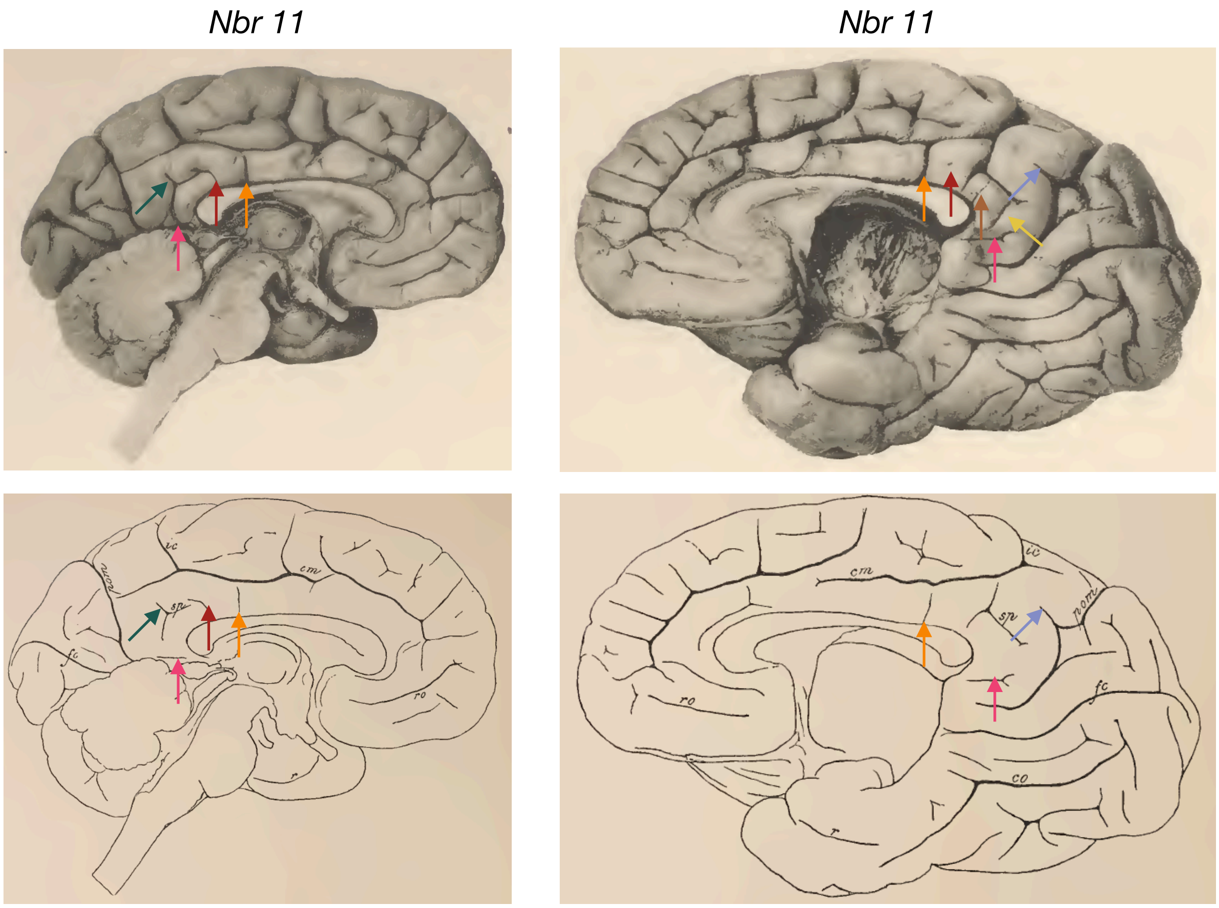


**Supplementary Figure 4. Manual PMC sulcal labels in the left and right hemispheres of postmortem chimpanzee brains.** Nine postmortem brains and their respective schematics adapted from Retzius' 1906 atlas[11] (images are in the public domain: https://www.law.cornell.edu/uscode/text/17/). PMC sulci are defined with arrows colored according to the key at the top of the figure. We do not mark the pos, spls, and mcgs.

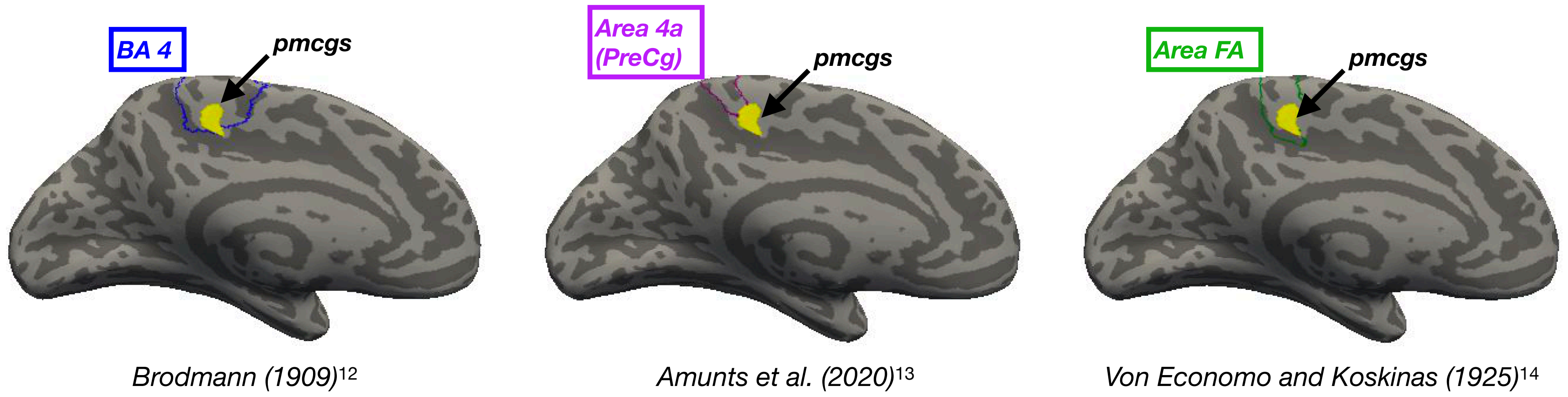


**Supplementary Figure 5. Premarginal branch of the cingulate sulcus (pmcgs) resides cytoarchitecturally outside of PMC.** An example inflated human left hemisphere with the pmcgs (yellow) identified relative to different cytoarchitectural definitions within its proximity: Area 4 (left; blue outline) defined by Brodmann[12], Area 4a (PreCg) (middle; purple outline) as defined by observer-independent methods by Amunts *et al.*[13], and Area FA (right; green outline) as defined by Von Economo and Koskinas[14]. In each case, the pmcgs resides outside of cytoarchitectonic PMC regions. This relationship also extends to chimpanzees[10]. Note that the areas around Area 4a have yet to be charted using modern, observer-independent techniques[13].

**Supplementary References**


1. Campbell, A. W. Histological studies on the localisation of cerebral function. (Cambridge University Press, 1905).
2. Vogt, C. & Vogt, O. Allgemeine Ergebnisse unserer Hirnforschung. (J.A. Barth, 1919).
3. Bailey, P. & von Bonin, G. *The Isocortex of Man*. (University of Illinois Press, 1951).
4. Gray, H. ANTOMY OF THE HUMAN BODY. *Ann. Surg.* **68**, 564 (1918).
5. Vogt, B. A., Nimchinsky, E. A., Vogt, L. J. & Hof, P. R. Human cingulate cortex: surface features, flat maps, and cytoarchitecture. *J. Comp. Neurol.* **359**, 490–506 (1995).
6. Petrides, M. Atlas of the Morphology of the Human Cerebral Cortex on the Average MNI Brain. (Academic Press, 2019).
7. Vogt, B. & Gabriel, M. Neurobiology of Cingulate Cortex and Limbic Thalamus: A Comprehensive Handbook. (Springer Science & Business Media, 2013).
8. Ono, M., Kubik, S. & Abernathey, C. D. *Atlas of the Cerebral Sulci*. (G. Thieme Verlag, 1990).
9. Vogt, B. Cingulate Neurobiology and Disease. (OUP Oxford, 2009).
10. Bailey, P., Bonin, G. V. & McCulloch, W. S. The isocortex of the chimpanzee. Vol. 440 University of Illinois Press. *Urbana* (1950).
11. Retzius, G. Cerebra simiarum illustrata. Das Affenhirn in bildlicher Darstellung. 304 (Stockholm,Centraldruckerei;, 1906).
12. Brodmann, K. Vergleichende Lokalisationslehre der Grosshirnrinde in ihren Prinzipien dargestellt auf Grund des Zellenbaues von Dr. K. Brodmann. (J.A. Barth, 1909).
13. Amunts, K., Mohlberg, H., Bludau, S. & Zilles, K. Julich-Brain: A 3D probabilistic atlas of the human brain's cytoarchitecture. *Science* **369**, 988–992 (2020).
14. von Economo, C. F. & Koskinas, G. N. Die cytoarchitektonik der hirnrinde des erwachsenen menschen. (J. Springer, 1925).
