# Supplementary material for: Sulcal morphology of posteromedial cortex substantially differs between humans and chimpanzees
Source: Commun Biol. 2023 Jun 1;6:586. doi: 10.1038/s42003-023-04953-5 (PMC10235074; doi:10.1038/s42003-023-04953-5)
Supplement: Supplementary file 3 — Reporting Summary [file 42003_2023_4953_MOESM3_ESM.pdf]

nature portfolio

Corresponding author(s): Kevin S. Weiner

Last updated by author(s): 5/15/2023

Reporting Summary

Nature Portfolio wishes to improve the reproducibility of the work that we publish. This form provides structure for consistency and transparency in reporting. For further information on Nature Portfolio policies, see our Editorial Policies and the Editorial Policy Checklist.

Statistics

For all statistical analyses, confirm that the following items are present in the figure legend, table legend, main text, or Methods section.

n/a	Confirmed	
☐	☒	The exact sample size (*n*) for each experimental group/condition, given as a discrete number and unit of measurement
☐	☒	A statement on whether measurements were taken from distinct samples or whether the same sample was measured repeatedly
☐	☒	The statistical test(s) used AND whether they are one- or two-sided<br>*Only common tests should be described solely by name; describe more complex techniques in the Methods section.*
☐	☒	A description of all covariates tested
☐	☒	A description of any assumptions or corrections, such as tests of normality and adjustment for multiple comparisons
☐	☒	A full description of the statistical parameters including central tendency (e.g. means) or other basic estimates (e.g. regression coefficient) AND variation (e.g. standard deviation) or associated estimates of uncertainty (e.g. confidence intervals)
☐	☒	For null hypothesis testing, the test statistic (e.g. *F*, *t*, *r*) with confidence intervals, effect sizes, degrees of freedom and *P* value noted<br>*Give P values as exact values whenever suitable.*
☒	☐	For Bayesian analysis, information on the choice of priors and Markov chain Monte Carlo settings
☒	☐	For hierarchical and complex designs, identification of the appropriate level for tests and full reporting of outcomes
☐	☒	Estimates of effect sizes (e.g. Cohen's *d*, Pearson's *r*), indicating how they were calculated

Our web collection on statistics for biologists contains articles on many of the points above.

Software and code

Policy information about availability of computer code

Data collection	MRI image processing and anatomical labeling was performed with the open source software: FreeSurfer v6.0.0 (https://surfer.nmr.mgh.harvard.edu/). FreeSurfer functions and custom code in Matlab (v2017a) were used to extract anatomical metrics.
Data analysis	All statistical tests were implemented using R (v4.0.1).

For manuscripts utilizing custom algorithms or software that are central to the research but not yet described in published literature, software must be made available to editors and reviewers. We strongly encourage code deposition in a community repository (e.g. GitHub). See the Nature Portfolio guidelines for submitting code & software for further information.

Data

Policy information about availability of data

All manuscripts must include a data availability statement. This statement should provide the following information, where applicable:

- Accession codes, unique identifiers, or web links for publicly available datasets
- A description of any restrictions on data availability
- For clinical datasets or third party data, please ensure that the statement adheres to our policy

Data and analysis pipelines used for this project will be made freely available on GitHub upon publication (https://github.com/cnl-berkeley/stable_projects). The colorblind-friendly color schemes used in our figures were created using the toolbox available at https://davidmathlogic.com/colorblind/. Requests for further information should be directed to the Corresponding Author, Kevin Weiner (kweiner@berkeley.edu).

Human research participants

Policy information about studies involving human research participants and Sex and Gender in Research.

Reporting on sex and gender	Participant gender was determined based on self-reporting and informed consent was obtained from all participants. Gender-based analyses were not performed as they were beyond the scope of the present study, which was focused purely on species-related differences.
Population characteristics	The anatomical T1 scans from 72 randomly-selected young adult participants from the Human Connectome Project (HCP) database (36 females, 36 males, aged 22-36) were used in the present study.
Recruitment	We did not recruit participants as we used freely available neuroimaging data from the HCP database: https://www.humanconnectome.org/study/hcp-young-adult/overview. Participants were randomly sampled from the HCP database blind to participant demographics.
Ethics oversight	HCP consortium data were previously acquired using protocols approved by the Washington University Institutional Review Board.

Note that full information on the approval of the study protocol must also be provided in the manuscript.

Field-specific reporting

Please select the one below that is the best fit for your research. If you are not sure, read the appropriate sections before making your selection.

☒ Life sciences ☐ Behavioural & social sciences ☐ Ecological, evolutionary & environmental sciences

For a reference copy of the document with all sections, see nature.com/documents/nr-reporting-summary-flat.pdf

Life sciences study design

All studies must disclose on these points even when the disclosure is negative.

Sample size	Young Adult Humans: 72 participants were sampled from the HCP database (https://www.humanconnectome.org/study/hcp-young-adult; see Van Essen et al., 2012).<br><br>Chimpanzees: 60 participants were sampled from an existing chimpanzee neuroimaging dataset (see Keller et al., 2009).<br><br>We chose our sample sizes based on those used in prior comparative work, which were robust enough to detect species-related differences in sulcal morphology (see Miller et al., 2020; Willbrand et al., 2022; Hathaway et al., 2022).
Data exclusions	No data was excluded in the present study.
Replication	We did not conduct analyses to replicate results in the present work; however, future work can seek to do so.<br><br>It is important to note that prior work has shown that a sample size of N = 20 is robust enough to detect neuroanatomical-related findings and encapsulate individual differences (e.g., Amunts and Zilles, 2015; Amunts et al., 2020; Miller et al., 2021; Voorhies et al., 2021; Yao et al., 2022; Weiner et al., 2014; Miller et al., 2020; Parker et al., 2023).
Randomization	All participants were randomly sampled from the larger datasets from which they were obtained.
Blinding	Blinding was not applicable for this study. However, all data were analyzed through a single pipeline to ensure minimization of influence from the researcher.

Reporting for specific materials, systems and methods

We require information from authors about some types of materials, experimental systems and methods used in many studies. Here, indicate whether each material, system or method listed is relevant to your study. If you are not sure if a list item applies to your research, read the appropriate section before selecting a response.

Materials & experimental systems

n/a	Involved in the study
☒	☐ Antibodies
☒	☐ Eukaryotic cell lines
☒	☐ Palaeontology and archaeology
☐	☒ Animals and other organisms
☒	☐ Clinical data
☒	☐ Dual use research of concern

Methods

n/a	Involved in the study
☒	☐ ChIP-seq
☒	☐ Flow cytometry
☐	☐ MRI-based neuroimaging

Animals and other research organisms

Policy information about studies involving animals; ARRIVE guidelines recommended for reporting animal research, and Sex and Gender in Research

Laboratory animals	The anatomical T1 scans from 60 in vivo chimpanzees (Pan Troglodytes; 37 female, 23 male) with ages between 9 and 51 were used in the present study. The chimpanzees were members of the colony housed at the Yerkes National Primate Research Center (YNPRC) of Emory University. Further data collection details regarding this sample are described in Keller et al., 2009.
Wild animals	This study did not involve wild animals.
Reporting on sex	Findings apply to both sexes. Sex was not considered in study design. Sex-based analyses were not performed as they were beyond the scope of the present study, which was focused purely on species-related differences.
Field-collected samples	This study did not involve samples collected in the field.
Ethics oversight	All methods were carried out in accordance with YNPRC and Emory University's Institutional Animal Care and Use Committee (IACUC) guidelines. Institutional approval was obtained prior to the onset of data collection. Chimpanzee MRIs were obtained from a data archive of scans collected prior to the 2015 implementation of U.S. Fish and Wildlife Service and National Institutes of Health regulations governing research with chimpanzees.

Note that full information on the approval of the study protocol must also be provided in the manuscript.

Magnetic resonance imaging

Experimental design

Design type	N/A
Design specifications	All participants underwent a high resolution T1-weighted structural imaging scan.
Behavioral performance measures	N/A

Acquisition

Imaging type(s)	T1-weighted MPRAGE (structural)
Field strength	3 Tesla
Sequence & imaging parameters	Young Adult Humans (HCP): Anatomical T1-weighted MPRAGE anatomical scans (TR=2400ms, TE=2.14ms, 0.8 × 0.8 × 0.8 mm voxels) were obtained in native space from the HCP database, along with outputs from the HCP modified FreeSurfer pipeline, for cortical morphometric analyses. Additional details on image acquisition parameters and image processing can be found in Glasser et al. (2013). Brain imaging data were collected on Siemens 3T scanners at multiple sites.<br><br>Chimpanzees: Here we briefly describe the scanning parameters that are described in more thorough detail in Keller et al., 2009. The T1-weighted magnetization-prepared rapid-acquisition gradient echo (MPRAGE) MR images were obtained using Siemens 3T Trio MR system (TR = 2300 ms, TE = 4.4 ms, TI = 1100 ms, flip angle = 8, FOV = 200 mm x 200 mm) at YNPRC in Atlanta, Georgia. Prior to reconstructing the cortical surface, each chimpanzee T1 was scaled to the size of the human brain. As described in Hopkins et al., 2017, within FSL, (1) the BET function was used to automatically strip away the skull, (2) the FAST function was used to correct for intensity variations due to magnetic susceptibility artifacts and radio frequency field inhomogeneities (i.e., bias field correction), and (3) the FLIRT function was used to normalize the isolated brain to the MNI152 template brain using a seven degree of freedom transformation (i.e., three translations, three rotations, and one uniform scaling), which preserved the shape of individual brains. Afterward, each T1 was segmented using FreeSurfer. The fact that the brains are already isolated, along with bias-field correction and size-normalization, greatly assisted in segmenting the chimpanzee brain in FreeSurfer. Furthermore, the initial use of FSL also has the specific benefit of enabling the individual brains to be spatially normalized with preserved brain shape. Lastly, the values of this transformation matrix and the scaling factor were saved for later use.
Area of acquisition	whole brain scan

Diffusion MRI ☐ Used ☒ Not used

Preprocessing

Preprocessing software	Freesurfer v6.0.0 was used for cortical surface reconstruction.
Normalization	N/A
Normalization template	N/A
Noise and artifact removal	All T1-weighted images were visually inspected for scanner artifacts. Each cortical surface reconstruction was visually inspected for segmentation errors.
Volume censoring	N/A

Statistical modeling & inference

Model type and settings	Linear mixed effects models (LME) were used to explore species-differences in the anatomical features of sulci in posteromedial cortex (PMC). Chi-squared tests and/or binomial logistic regression general linear models (GLM) were implemented to compare the sulcal types of the marginal ramus of the cingulate sulcus and incidence rates PMC sulci, respectively, between species.
Effect(s) tested	A LME with predictors of hemisphere and species, as well as their interaction terms, was used to test for species-differences in the amount of PMC buried in sulci. ANOVA F-tests were applied to the model.<br><br>We tested the influence of species and hemisphere on the probability of a sulcus to be present with binomial logistic regression GLMs. For each statistical model, species (human, chimpanzee) and hemisphere (left, right), as well as their interaction, were included as factors for presence [0 (absent), 1 (present)] of a sulcus.<br><br>To compare whether the incidence of the variable PMC sulci in chimpanzees related to one another, we ran binomial logistic regression GLMs for each variable PMC sulcus [0 (absent), 1 (present)] with the other sulci as factors, while also including an interaction with hemisphere for each sulcus. ANOVA Chi-squared tests were applied to each GLM.<br><br>We quantitatively determined whether the incidence rates of the four mcgs types differed by species, as well as between hemispheres for each species, with Chi-squared tests.<br><br>To assess whether the depth and surface area of PMC sulci differed between chimpanzees and humans, for both morphological features, we ran a LME with predictors of sulcus, hemisphere, and species, as well as their interaction terms. We repeated the prior analysis, exchanging the factor of PMC sulci for the mcgs branch (main branch, dorsal branch, side branch) to test for species-differences in mcgs anatomy. ANOVA F-tests were applied to each model.

Specify type of analysis: ☐ Whole brain ☒ ROI-based ☐ Both

Anatomical location(s)	The sulci within posteromedical cortex (our ROI) were defined anatomically using the most recent and comprehensive sulcal atlas (Petrides, 2019) and other studies (Willbrand et al., 2022; Borne et al., 2020; Margulies et al., 2009; Vogt et al., 1995) as references.
Statistic type for inference (See Eklund et al. 2016)	N/A
Correction	Tukey's method to correct for multiple comparisons.

Models & analysis

n/a	Involved in the study
☒	☐ Functional and/or effective connectivity
☒	☐ Graph analysis
☒	☐ Multivariate modeling or predictive analysis
